# Unexplained long-term increase in intraocular pressure during the treatment of macular disease with intravitreal anti-VEGF

**DOI:** 10.1007/s00417-025-07100-4

**Published:** 2026-01-10

**Authors:** Lisa-Marie Eisenrauch, Yaser Abu-Dail, Cristian Munteanu, Elias Flockerzi, Berthold Seitz, Alaa Din Abdin

**Affiliations:** 1 Department of Ophthalmology, Saarland University Medical Center UKS, Homburg/Saar, Germany; 2 Department of Ophthalmology, Saarland University Medical Center UKS, Kirrberger Straße, Bldg. 22, 66421 Homburg/Saar, Germany

**Keywords:** Intraocular pressure (IOP), Anti-VEGF therapy, Macular diseases, Intravitreal injection

## Abstract

**Purpose:**

This study aimed to investigate the incidence and timing of unexplained intraocular pressure (IOP) increase over time following the administration of various intravitreal anti-vascular endothelial growth factor (anti-VEGF) agents for macular diseases.

**Patients and methods:**

This retrospective study included 2611 eyes treated multiple times with one or more different anti-VEGF agents between 2016 and 2022. An IOP increase of ≥ 25 mmHg was classified as pathological. We analyzed the incidence rates, the timing of the IOP increase during the administered therapy and the thickness of the retinal nerve fiber and ganglion cell layer over two years in eyes with IOP increase.

**Results:**

A total of 50 eyes (1.9%) from 48 patients experienced an increase in IOP during anti-VEGF therapy. In 15 eyes, the increase was attributable to other ocular diseases (such as neovascular glaucoma, uveitis, or endophthalmitis), and these eyes were therefore excluded from further analysis. In contrast, 35 eyes (1.3%) from 33 patients with an average age of 68.4 ± 10,0 years, developed an unexplained increase in IOP up to an average of 27 [25–45] mmHg, typically after 12 [1–35] weeks. 97% of the affected eyes had no history of glaucoma. The baseline IOP was 16 [12–20] mmHg. In 24 eyes (68%), short-term topical therapy led to adequate IOP regulation. For 10 eyes (29%) with persistently elevated IOP, continued topical antiglaucoma therapy was required, while 2 eyes required surgical intervention. The average thickness of the retinal nerve fiber layer and the ganglion cell layer did not change significantly over two years.

**Conclusion:**

An increased IOP can occur during the course of anti-VEGF therapy in normotensive eyes. This phenomenon can be conservatively controlled in 94% of cases.

## Key Messages

*****What is known***:**

- An increase in intraocular pressure can be observed both immediately after intravitreal injection and in the longer term during the course of therapy.

*****What is new***:**

- The long-term increase in intraocular pressure during the course of therapy with various intravitreal anti-vascular endothelial growth factors can be considerable, but is often only temporary.
- This phenomenon can be controlled conservatively in most cases.

## Introduction

Various diseases of the macula, such as age-related macular degeneration (AMD), retinal vein occlusion (RVO) or diabetic macular edema (DME) are among the leading causes of blindness worldwide [1, 2]. A contemporary treatment for these diseases is the introduction of intravitreal injections (IVI) with anti-angiogenic agents. These include therapy with anti-vascular endothelial growth factors (anti-VEGF therapy) with different agents (bevacizumab, aflibercept, ranibizumab, brolucizumab and faricimab). Studies have shown that they can significantly improve vision and slow the progression of macular diseases [3].

Although this is a revolutionary treatment option [4], potential side effects have been reported, particularly in relation to increasing intraocular pressure (IOP). An increase in IOP can be observed both immediately after intravitreal injection and in the longer term during the course of therapy [5]. This phenomenon poses a significant challenge, as persistently elevated IOP increases the risk of developing glaucoma. Subsequently, it can lead to irreversible damage to the optic nerve and a deterioration in vision [6].

The exact mechanism of action by which anti-VEGF drugs affect IOP is not yet fully understood. While a plausible link between anti-VEGF therapy and a short-term increase in IOP immediately after drug administration has been demonstrated [7], the long-term effect of multiple anti-VEGF injections remains controversial [8].

The **purpose** of the present study is to determine the incidence rates, timing and therapy options of unexplained increases in IOP over time following the administration of various intravitreal anti-VEGF agents for the treatment of macular diseases.

## Patients and methods

### Description of study groups

The data were collected retrospectively from 01.01.2016 to 30.04.2022 and included 2611 eyes. The study took place at the Intravitreal Injections Center of the Department of Ophthalmology at Saarland University Medical Center (UKS) in Homburg [9].

The participating patients were identified using the FIDUS patient database (Arztservice Wente GmbH, Darmstadt).

The inclusion criteria were the presence of macular disease mostly neovascular age-related macular degeneration (nAMD), diabetic macular edema (DME) and retinal vein occlusion (RVO) requiring intravitreal treatment. Only patients under anti-VEGF therapy were further analyzed in combination with an IOP increase. Patients with existing glaucoma or a positive family history of glaucoma were also included in the study. In these cases, critical attention was paid to ensuring that IOP pressure values were in the range between 10 mmHg and 21 mmHg, as was the case with the other patients without prevalent glaucoma.

Patients under 18 years and patients with an **explained** IOP increase like neovascular glaucoma, uveitis, or endophthalmitis were excluded from the study because these conditions are known to cause an increase in intraocular pressure independently of anti-VEGF therapy.

Intravitreal medication with any type of steroid was also excluded. The macular diseases were diagnosed by means of fundoscopy, optical coherence tomography and fluorescein angiography. All examinations were performed before IVI. IVIs included various anti-VEGF agents (bevacizumab, aflibercept, ranibizumab, brolucizumab), which were used once or several times.

Once an increase in IOP was recorded, we documented the time between the first administration and the IOP increase, and which drug was administered at that time. The threshold for describing ocular hypertension was 25 mmHg [10].

The treatment options in the study included both conservative eye drops and various operations. The short-term topical therapy to reduce IOP was a series of brimonidine eye drops (5x/h). Further elevated IOPs were lowered with anti-glaucoma eye drops. Cyclophotocoagulation (CPC) and trabeculectomy (TE) were performed to surgically regulate the IOP. Subsequently, it was evaluated whether the therapy had a positive effect on the IOP. IOPs below the defined limit were considered to be well controlled [10, 11].

## Outcome measures for patients with unexplained IOP increase

**Medical history**: included a query about arterial hypertension and diabetes mellitus (DM). The ophthalmologic history included identifying the diseased eye and the underlying disease, previous pars plana vitrectomy (PPV), cataract surgery, trauma or evidence of existing primary glaucoma. The family history of glaucoma and the lens status were also recorded.

The following parameters from the IVI center database were documented for baseline on the day of the first elevated of IOP and after 6, 12 and 24 months:

- Intraocular pressure (IOP).
- Thickness of the retinal nerve fiber layer (RNFL).
- Thickness of the retinal ganglion cell layer (RGCL).
- Cup-disc ratio (CDR): based on the medical examination.
- Therapy → conservative, number of eye drops.
- Therapy → Cyclophotocoagulation, filtering glaucoma surgery.

## Results

**Baseline demographic data**: The underlying diagnoses included.

- neovascular age-related macular degeneration (nAMD) in 29% of eyes.
- diabetic macular edema (DME) in 38%,
- retinal vein occlusion (RVO) in 24%,
- postoperative macular edema (PME) in 6%,
- choroidal neovascularization upon rare sight problems (rCNV) in 3%.

Arterial hypertension was present in 66% of patients and 51% suffered from DM.

Glaucoma was diagnosed in 3% of cases, and 3% of patients also reported a positive family history of glaucoma.

In terms of lens status, 26% of eyes were phakic and 74% had pseudophakia.

A previous pars plana vitrectomy (PPV) was performed in 11% of the patients.

In terms of initial anti-VEGF therapy, patients received.

- 74% Bevacizumab.
- 11% Aflibercept.
- 15% Ranibizumab.

A switch of therapy took place in 20% of eyes, with 17% of patients switching from bevacizumab to aflibercept and 3% switching from aflibercept to ranibizumab (Table 1).

**Table 1:** Baseline characteristics for eyes with unexplained increase in intraocular pressure (*n* = 35)

Baseline characteristics	Number	%
Diagnosis (AMD: DME: RVO: PME: rCNV)	10:14:8:2:1	29:40:22:6:3
Arterial hypertension	23	66
Diabetes mellitus	18	51
Glaucoma	1	3
Family history of Glaucoma	1	3
Lens status (Phakic: pseudophakic)	9:26	26:74
Previous Pars Plana Vitrectomy	4	11
Baseline intravitreal agent (bevacizumab: aflibercept: ranibizumab)	26:4:5	74:11:15
Current intravitreal agent (bevacizumab: brolucizumab: aflibercept: ranibizumab)	24:1:5:5	69:3:14:14
History of therapy switch (first: second)	7 (6:1)	20 (17:3)

*nAMD* Neovascular age-related macular degeneration, *DME* Diabetic macular edema, *RVO* Retinal vein occlusion, *PME* Postoperative macular edema, *rCNV* choroidal neovascularization by other causes

### Eyes with an unexplained increase in IOP

In this retrospective analysis, 35 eyes showed an unexplained increase in IOP.

At the time of the IOP increase, anti-VEGF therapy was administered in.

- 69% of cases with bevacizumab.
- 14% of cases with ranibizumab,
- 14% of cases with brolucizumab,
- 3% of cases with aflibercept.

**Intraocular pressure (IOP)** fluctuated significantly over the 24-month observation period following injection. At the time point of the first elevation (0 month), the IOP of 29 ± 6 mmHg (mean ± standard deviation) was significantly higher than the initial value of 16 ± 4 mmHg (baseline value). After 6 months, the IOP normalized to 19 ± 6 mmHg and remained stable over the following observation periods (12 months: 16 ± 4 mmHg, 24 months: 17 ± 4 mmHg). In our study, anti-VEGF treatment was continued after medication had reduced elevated intraocular pressure to normal levels. Patients were then closely monitored to detect any renewed increases in pressure at an early stage.

**The cup-disc ratio (CDR)** remained statistically stable over the entire period, with minimal fluctuations from 0.4 ± 0.2 at baseline to 0.5 ± 0.4 after 24 months.

**The retinal nerve fiber layers (RNFL)** and **the retinal ganglion cell layer (RGCL)** also showed no significant changes. RNFL thickness in the 3-mm zone ranged between 36 ± 33 μm and 34 ± 12 μm by month 24. Similarly, RNFL thickness in the 6-mm zone, which ranged between 36 ± 13 μm and 33 ± 11 μm after 24 months.

RGCL thickness remained statistically stable, with a value in the 3-mm zone of 35 ± 13 μm at baseline and 35 ± 17 μm at 24 months. In the 6-mm zone, the value ranged from 29 ± 70 μm to 29 ± 10 μm after 24 months. Finally, the average thickness of the RNFL and RGCL did not change statistically significantly over the observation period of two years follow-up (Table 2).

**Table 2:** Follow-up for eyes with unexplained increase in intraocular pressure (*n* = 35)

	IOP (mmHg)	Cup-Disk Ratio	RNFL 3 mm (µm)	RNFL 6 mm (µm)	RGCL 3 mm (µm)	RGCL 6 mm (µm)
Baseline	16 ± 4	0.4 ± 0.2	36 ± 33	36 ± 13	35 ± 13	29 ± 7
0 M	29 ± 6	0.5 ± 0.2	37 ± 32	42 ± 21	36 ± 14	30 ± 8
6 M	19 ± 6	0.5 ± 0.3	39 ± 54	40 ± 23	30 ± 16	30 ± 9
12 M	16 ± 4	0.5 ± 0.3	28 ± 15	36 ± 14	34 ± 15	30 ± 8
24 M	17 ± 4	0.5 ± 0.4	34 ± 12	33 ± 11	35 ± 17	29 ± 10

*IOP* intraocular pressure, RNFL retinal nerve fiber layer, RGCL retinal ganglion cell layer

## Treatment of elevated intraocular pressure (IOP)

- In 23 eyes (68%), a single acute increase in IOP was detected; thus, adequate IOP control was achieved with short-term topical therapy (a series of brimonidine eye drops, 5×/hour).
- In 12 eyes (32%), IOP increased again after continuing anti-VEGF treatment.
  - ✓ In a further 10 eyes (28%), topical antiglaucoma therapy had to be continued.
  - ✓ Surgical intervention was required in 2 eyes (4%), including one case treated with CPC and another with TE.

In our cohort, neither the cumulative number of injections, injection frequency, nor treatment switching correlated with the severity of IOP elevation, and no significant differences were observed between the individual anti-VEGF agents (Table 3).

**Table 3:** Association between treatment-related factors and the severity of IOP elevation

Factors		IOP at diagnosis (mmHg)	*p* value
Switching	**Monotherapy** (***n*=28**)	29.2±5.4	0.34
	**After switching **(***n*=7**)	31.7±7.9	
Number of IVIs	**<10 **(***n*=24**)	29.7± 5.7	0.99
	**>10** (***n*=11**)	29.7±6.8	
Agent	**Bevacizumab** (***n*=24**)	28.1±3.4	0.28
	**Aflibercept** (***n*=5**)	32.0±5.5	
	**Ranibizumab** (***n*=5**)	30.8±8.6	

*IOP* intraocular pressure, *IVI* intravitreal injections

## Discussion

This study assessed the incidence rate and time course of an increase in intraocular pressure after administration of different intravitreal anti-VEGFs for the treatment of macular diseases and demonstrated that therapy with anti-VEGF drugs may be associated with a significant, but often transient, increase in intraocular pressure.

The frequency of an IOP increase following anti-VEGF therapy varies within the studies, with an average between 3.5% and 13% of a sustained increase in pressure reported during the course of therapy [12, 13]. These discrepancies may be due to different study designs and cohorts, particularly differences in the definition of what was considered a “significant” IOP elevation [10, 14]. In our study, the incidence rate was 1.9%. This corresponded to 50 of a total of 2611 included eyes. A significant IOP increase was based on the definition suggested by Malclès et al. [10]. In line with the SAFODEX study by Malclès et al., we defined pathological intraocular pressure elevation as an absolute IOP of ≥ 25 mmHg or an increase of ≥ 10 mmHg from baseline. The SAFODEX authors based their definition on the classic classification of steroid-induced pressure responders according to Becker and Armaly, according to which pressure increases of 6–15 mmHg (intermediate responder) or > 15 mmHg (high responder) are considered clinically relevant. The absolute threshold of 25 mmHg also corresponds to the definition of pathological hypertension commonly used in numerous intravitreal studies [10].

Although anti-VEGF preparations were used in our work rather than intravitreal steroids, these IOP thresholds are also appropriate for our research question. They represent generally accepted, clinically relevant thresholds for reliably detecting treatment-associated pressure spikes, regardless of the mechanism of action. Using the same criteria as in SAFODEX also allows for better comparability of our results with existing publications on intravitreal therapies.

Our goal was therefore to record the rate of clinically relevant ocular hypertension and not every short-term pressure fluctuation after the injection. A possible reason for the lower percentage compared to the previously mentioned studies may be due to the strict inclusion and exclusion criteria in connection with our study design. The following results are intended to provide an overview of the frequency of IOP increase over time.

## Persistent IOP increase

A persistent IOP increase over time is a potentially serious side effect. The extent to which intravitreal drug administration causes persistent IOP increase, even over a period of several years, remains an underestimated issue. Several studies reported that between 4% and 15% of patients had a persistent IOP increase in 9 to 24 months after an injection, while in about the same number of studies there was no long-term change between 1 and 36 months after the first IVI [5].

Furthermore, the study by Nanji et al. [15] suggests that there is no clear difference between patients with nAMD, RVO or DME and the control group in terms of the IOP increase after 12 and 24 months. This study also showed a mean normotensive IOP of 16 ± 40 at 12 months and 17 ± 4 at 24 months.

Furthermore, there were no differences in regards to which anti-VEGF preparations were used for more frequent injections, as reported in some other studies [16].

### Treatment of IOP increase

Therapy with IOP-lowering medication: The use of topical medications is a possible first-line treatment for elevated IOP caused by anti-VEGF injections [17]. Various studies have investigated the efficacy of these forms of therapy, particularly in relation to acute and short-term IOP elevation.

Surgical therapy: Cases of therapy-refractory IOP increase may require surgical therapy [18], Williams and Argáez [19] also reported that one in 127 patients (0.6%) with nAMD required surgical intervention in the form of trabeculectomy to control the permanently elevated IOP. This is also reflected in our study. Only 2 of the 35 eyes (4%) with elevated IOP had to undergo surgery as a control measure. One eye was treated with TE, the other with CPC.

Monitoring of IOP over time: The possibility of a long-term increase in IOP after IVI should be critically evaluated in eyes with an increased risk of glaucoma or existing glaucoma, particularly in advanced stages. In these cases, close monitoring of IOP is required [20]. Specifically with regard to ranibizumab, 1125 eyes were examined in the study by Bakri et al. [21]. These eyes showed a more frequent increase in IOP compared to the baseline value than eyes that underwent sham treatment.

### Effect on retinal nerve fiber layer, ganglion cell layer and CDR

In patients treated with anti-VEGF drugs such as ranibizumab, bevacizumab and aflibercept over a two-year period, there was no significant reduction in the thickness of the RNFL. However, local reductions in the thickness of the RGCL were observed in some areas. These changes may result from direct RGCL damage caused by treatment of the underlying disease [22]. Some studies support these findings, while others report contrasting results. Martinez-de-la-Casa et al. and Valverde-Megías et al. [23, 24] showed a reduction in RNFL thickness, while Sobaci et al. found no significant changes in RNFL. Comparisons between the drugs ranibizumab, aflibercept and bevacizumab showed no significant differences on RNFL over two years [25]. In our study, the average thickness of the RNFL also did not change statistically significantly over the two-year period in the 35 patients with an IOP increase.

The effect of anti-VEGF therapy on the GCL was also investigated in studies, although the results in this regard also varied. In a study by Inan et al. [26], there was a significant reduction in the RGCL thickness over a period of one year. In comparison, Makri et al. [27] and Cao et al. [28] reported that there was no significant thinning of the layer thickness over a longer observation period of one and three years, respectively. In our study, there was no statistically significant change in RGCL over the two-year follow-up.

The main limitations of this study were the retrospective nature and the monocentric study design, which limits external validity. Treatment strategies, the selection of anti-VEGF drugs used, and patient characteristics may vary between different centers, thereby influencing the frequency and management of IOP elevation. At the same time, the large number of cases (over 2,500 eyes) examined under uniform diagnostic and therapeutic conditions strengthens the internal validity of the study, as standardized diagnostics, identical measuring instruments, and consistent follow-up reduce variability and improve the comparability of the data.

In addition, dependence on clinical examinations to assess parameters such as the CDR must be considered as a further limitation.

In conclusion, an increased IOP can occur during the course of anti-VEGF therapy in normotensive eyes. This phenomenon can be conservatively controlled in 94% of cases.
